# Adherence to exercise rehabilitation programmes in stroke survivors: a scoping review

**DOI:** 10.1136/bmjsem-2024-002102

**Published:** 2025-09-10

**Authors:** Nicole Gwynne-Mayer, Avril E Drummond, Jennie Hancox, Wafa Alrubaia, Ian M Taylor

**Affiliations:** 1 School of Sport, Exercise and Health Sciences, Loughborough University, Loughborough, UK; 2 University of Nottingham Faculty of Medicine and Health Sciences, Nottingham, UK

**Keywords:** Exercise, Intervention, Review, Psychology, Behaviour

## Abstract

**Objectives:**

To identify how exercise adherence is reported in research which focuses on enhancing cardiorespiratory and muscular fitness in stroke survivors.

**Design:**

Systematic scoping review.

**Data sources:**

Scopus, PubMED/MEDLINE, Cochrane Central Register of Controlled Trials, Cumulative Index to Nursing and Allied Health Literature, PsycINFO/PsycARTICLES and Web of Science.

**Eligibility criteria for selecting studies:**

The review sought published reports involving a cardiorespiratory or resistance training exercise intervention for people who experienced a stroke ≥6 months prior to participation. Reports were screened by two reviewers and were assessed for methodological quality using the Mixed Methods Appraisal Tool.

**Results:**

37 reports (randomised trials=30, interventions without control=5, non-randomised trial=1, dose escalation trial=1; total n=1347 participants) out of 6732 records met the inclusion criteria. 29 reports (78%) documented intervention adherence by describing participant completion rates, indicating 65–100% adherence. *Exercise session* adherence within intervention was identifiable in 16 reports (43%), 12 of which recorded session attendance, signifying 61–100% adherence. There were also measures of the intensity or duration participants sustained during sessions in 14 studies. Only one study measured postintervention (ie, at least 3 months after intervention completion) exercise adherence, and two studies actively supported participants’ exercise motivation.

**Conclusion:**

Adherence is commonly measured by intervention completion rates. Despite its importance for intervention effectiveness, less than half of studies measured exercise session adherence within interventions. Future research should address this limitation to better understand how stroke survivors engage with exercise.

**WHAT IS ALREADY KNOWN**

- Previous reviews specifically on exercise adherence after a stroke have focused on reports which examine adherence as an outcome measure. However, this may have narrowed the wider understanding of how intervention studies appraise exercise adherence in stroke recovery.

**WHAT ARE THE NEW FINDINGS**

- There are important differences between adherence to the intervention, defined by participants’ completion frequencies and the degree to which participants adhere to exercise within an intervention, such as the quality of exercise within the intervention.
- Most reports document intervention completion rates, though only a small number of reports provided information which could be used to show participants’ quality of adherence to exercise within interventions or longer-term engagement with exercise.

## Introduction

 Approximately a quarter of people will experience a stroke at some point in their lives.^1^ Not only is stroke a major cause of death worldwide, but people who survive can experience health problems, such as loss of mobility, pain, fatigue, difficulties with cognitive functioning, impaired speech, personality changes and poorer mental health.^2^,^4^ As such, most people who have experienced a stroke require extensive rehabilitation.

Enhancing physical movement for people with stroke is important due to its association with reducing stroke reoccurrence and mortality.^5^ Exercise is a subcategory of physical activity that is planned and repetitive with an objective to enhance or maintain physical fitness.^6^ Exercise after a stroke has the potential to widely benefit stroke survivors in their physical and psychological recovery because it helps long-term muscular development and physical functioning,^6^,^9^ supports executive functioning and cognition,^10 11^ reduces fatigue, improves sleep and increases mental well-being.^12^ The recent UK National Clinical Guidelines for Stroke, published by the Royal College of Physicians, also recommends engagement with exercise and specifically endorses high-intensity cardiorespiratory and strength training for recovery and the prevention of further strokes.^13^

While the benefits from engaging with exercise are clear, people must adhere to programmes for them to be realised. Adherence is defined by the WHO as the extent to which a person follows healthcare instructions.^14^ In rehabilitation settings, adherence is routinely determined by whether a person has completed (ie, finished or dropped out of) a prescribed therapeutic programme (also described as retention).^15^ This approach typically leads to poor adherence to exercise being documented,^16^ but these low rates may simply be a reflection on adherence measurement and under-reporting rather than actual uptake of exercise.^17^,^20^ Other ways that exercise adherence has been inconsistently reported include using class attendance or duration and intensity of exercising.^21^ Sometimes, these measures have been compared with varying target rates or cut-off points, such as attendance at two-thirds of prescribed exercise sessions being classified as adherence.^21^ Overall, it is difficult to determine adherence rates due to varied measures and inconsistent reporting; something which has been highlighted by previous reviews of poststroke adherence to home exercise or various physical activity programmes.^18 22^

The present review aims to broadly explore adherence to cardiorespiratory and resistance training for people ≥6 months after stroke. It is important to consider adherence in these individuals because they typically have long-term outcomes of stroke.^23^ Previous reviews have identified how adherence is incorporated into randomised controlled trials of exercise training or aerobic and resistance training.^24 25^ This review seeks to evaluate adherence in all exercise-related interventions aimed at people with stroke, not limited to studies that assessed adherence as an explicit outcome measure or only randomised controlled trials. A scoping review was considered appropriate because a wide and diverse group of studies is not suited to other types of review.^26 27^ This scoping review has the following research questions:

1. How is adherence defined and measured in exercise interventions aimed at enhancing cardiorespiratory and muscular fitness for stroke survivors at least 6 months after stroke?
2. What are the adherence rates to cardiorespiratory and resistance training interventions for stroke survivors at least 6 months after stroke?
3. What methods are employed to support stroke survivors’ adherence to cardiorespiratory and resistance training interventions?

## Methods

No ethical approval was required for this scoping review. This review followed the Preferred Reporting Items for Systematic reviews and Meta-Analyses Extension for Scoping Reviews (PRISMA-ScR)^28^ guidelines. The PRISMA-ScR checklist can be seen in online supplemental data 1.

### Protocol and registration

The review protocol can be found at https://osf.io/f5u2t which was published after record collection, during the final report screening for the review.

### Eligibility criteria

We sought reports published in a peer-reviewed journal and written in the English language. As shown in table 1, reports were subject to the following primary selection criteria: (a) population: stroke survivors who had their stroke at least 6 months previously; (b) intervention: an exercise intervention within the rehabilitative stage of people’s recovery from stroke. Exercise must be carried out without physical assistance from an external source (eg, instructor or machine) and aim to enhance one or more elements of health-related fitness, specifically cardiorespiratory endurance, muscular endurance or muscular strength^29^; (c) outcome: one or more measures relating to cardiorespiratory endurance, muscular endurance, or muscular strength; (d) *study design*: trials including both randomised and non-randomised designs, within and between-subject designs, as well as interventions without control groups.

**Table 1:** The inclusion criteria of analysed reports

Area	Included	Excluded
Study type	Randomised controlled trials Non-randomised trials Cross-over/within- subjects design Intervention studies without control groups	Case studies Qualitative studies Reviews, meta-analyses Commentaries or similarsr similar Book chapters Unpublished articles
Participants	Aged ≥18 years old adults who had a stroke at least 6 months prior to the study, when their stroke symptoms are more likely to be stable.	Mixed participant pool of diseases outside of stroke if the results do not separate these different populations Patients with subarachnoid haemorrhage or subdural haematoma, as these patients undergo different treatments, as well as individuals who have experienced a transient ischaemic attack
Exercise intervention	Exercise programmes post-stroke Any physical or geographical location Clear unassisted exercise intervention which replicates accessible exercise to most people Purpose of exercise must relate to a cardiorespiratory or muscular fitness component of exercise such that an outcome measure must include testing that relates to one of the following: Cardiorespiratory endurance. Muscular endurance. Muscular strength.	Use of equipment which is not commercially available Assisted exercise including methods such as robotics, electrical stimulation, drug/pharmaceutical use, interference with free movement of the body such as constrained movement therapy, mechanically altered surfaces (balance boards), altered sensory feedback using virtual reality, video gaming, mirror therapy, action observation therapy, as well as the use of feedback which scopes beyond everyday personal monitoring including feedback from a therapist to assist effort input and advanced biofeedback

### Information sources

Databases searched for published reports included Scopus, PubMED/MEDLINE, Cochrane Central Register of Controlled Trials, Cumulative Index to Nursing and Allied Health Literature, PsycINFO/PsycARTICLES and Web of Science. Start dates were not specified; therefore, the searches ran for ‘all years’ included in each of the databases.

### Search

Online supplemental data 2 display the full search strategy and limiters applied to databases. Three search term groupings ‘Stroke’, ‘Rehabilitation’ and ‘Exercise’ were used in combination. Searches of terms targeted report titles and abstracts.

### Selection of sources of evidence

The search results were exported into Covidence systematic review software^30^ by NG-M. Initial title and abstract screening of reports was conducted separately by NG-M and WA following the inclusion criteria. Conflicts were resolved by IMT who made the final decision whether the reports met the eligibility criteria. Reports which proceeded to full-text review were also screened for final analysis with the same arrangement.

### Data charting process

The form to extract data from each study was developed based on the Population, Intervention, Comparison and Outcomes framework.^31^ Data were extracted and analysed independently by NG-M and subsequently reviewed in collaboration with IMT.

### Data items

Data extracted from reports included basic study information (authors, year of publication); participant characteristics (sample size, inpatient or out-patients); exercise type; exercise programme characteristics (eg, setting, duration, frequency, supervised/unsupervised); study design; participant recruitment; if adherence was measured; how adherence was measured; adherence outcome statistics, use of strategies to enhance adherence; information for assessment of the risk of bias.

### Critical appraisal of individual sources of evidence

The Mixed Methods Appraisal Tool (MMAT)^32^ was used to appraise the quality of reports included in the review. Assessment was done by NG-M. The MMAT is a useful tool to evaluate the quality of reports with different methods. Online supplemental data 3 show the findings of the MMAT assessment for the articles included in this review. All questions from the MMAT were used on reports.

### Synthesis of results

A narrative synthesis of the key findings from the reports and summary table was developed. The synthesis was structured around how adherence was operationalised and measured, adherence rates and degree of support provided to participants.

### Equity, diversity and inclusion statement

The research team involves early career (two) and experienced (three) researchers, consisting of four women and one man. The authors’ research disciplines involve sport, exercise and health sciences and are based in the UK. The research population includes adults who have had a stroke, and it is recognised that the findings of this review may not be generalisable to other populations.

### Patient and public involvement

Patients and the public were not involved in the design, development and dissemination of this review. The review findings are intended for researchers, clinicians and policymakers.

## Results

### Selection of sources of evidence

**Figure 1:**
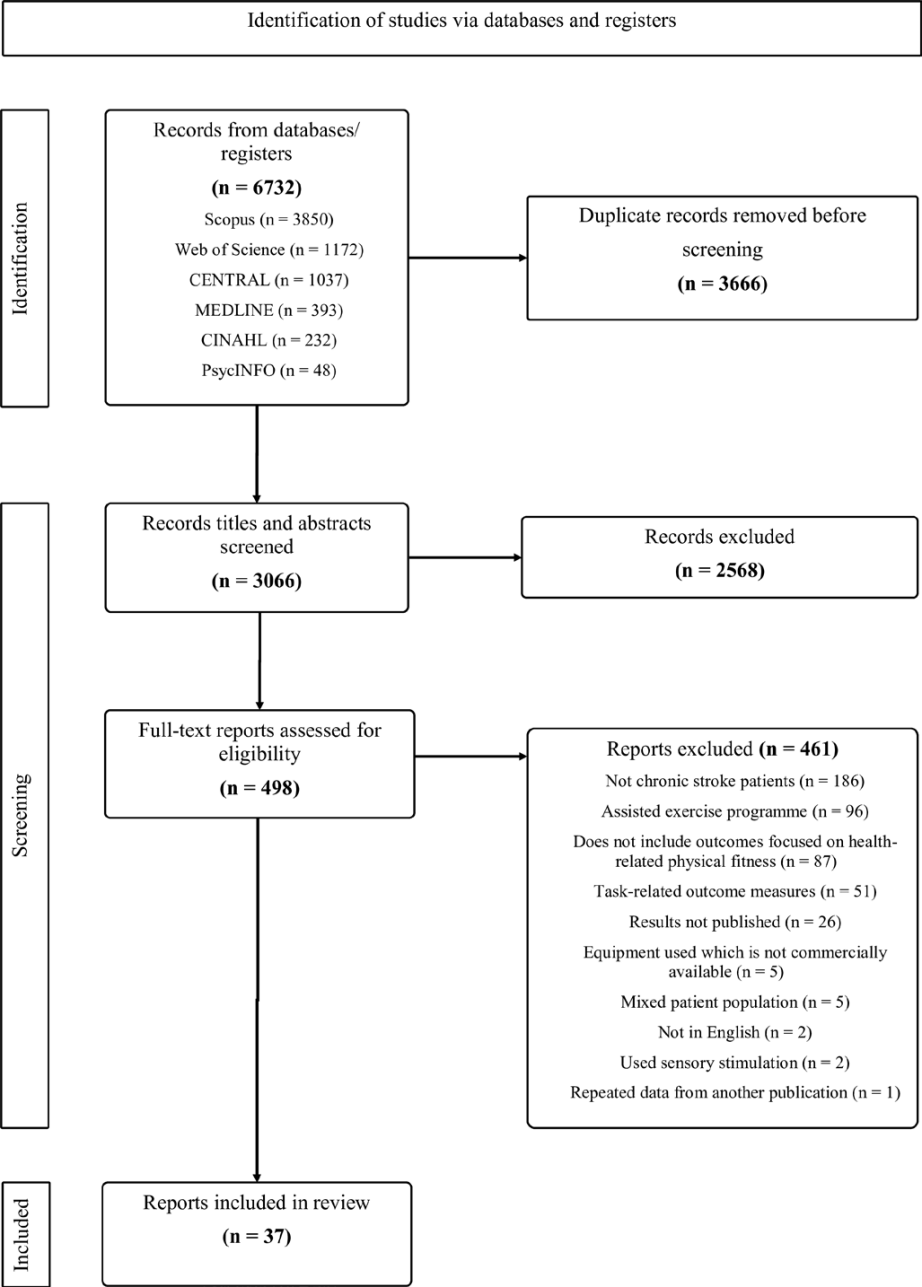
A Preferred Reporting Items for Systematic reviews and Meta-Analyses flow diagram of the report screening process.

Databases were searched on 12 February 2024, yielding 6732 records. After the automatic removal of duplicates on the Covidence software, other duplicate records were removed manually by NG-M. Abstracts of the remaining 3066 articles were reviewed, and records were excluded from full-text review based on study design, diagnosis of stroke and not involving an exercise intervention. After the first stage of screening, 498 records met the criteria for full-text review. A decisive part of the full-text screening process was whether the exercise intervention targeted cardiorespiratory endurance, muscular endurance or muscular strength (ie, was consistent with the definition of ‘exercise’), and included an outcome measure that assessed one of these fitness outcomes. It was also important that exercise programmes did not require physical assistance of movement or advanced feedback which scopes beyond everyday personal monitoring (such as virtual reality use or advanced biofeedback monitoring). These exclusions were made because these forms of exercise are likely to have markedly different adherence-related issues, compared with more common exercise interventions. The review included only participants who were 6 months post stroke. This stage of screening yielded 37 reports (total n=1347) shown in table 2 which met the full inclusion criteria. In the case that two reports presented one study, both reports were considered for inclusion in the review. However, provided that both reports shared the same data, the most recent publication was selected for the final analysis. Figure 1 shows the report selection flow chart.

**Table 2:** Included reports in the review

Lead author	Year	Country	Design
Bang	2016^44^	Republic of Korea	Randomised control trial
Boyne	2015^45^	United States	Randomised crossover trial
Boyne	2016^46^	United States	Randomised control trial
Chun	2015^35^	Korea	Randomised control trial
Cramp	2006^47^	United Kingdom	Pre-post trial
Dite	2015^48^	Australia	Dose escalation trial
El-Tamawy	2021^49^	Egypt	Randomised control trial
Fathi	2022^50^	Iran	Randomised control trial
Flansbjer	2012^33 34^	Sweden	Randomised Control Trial 4 year follow-up
Fonseca	2022^51^	Brazil	Randomised control trial
Hashidate	2011^52^	Japan	Pre-post trial
Jin	2013^53^	China	Randomised control trial
Kim	2017^54^	Republic of Korea	Randomised Control Trial
Kim, K	2014^55^	Republic of Korea	Randomised Control Trial
Lattouf	2021^56^	Lebanon	Randomised Control Trial
Linder	2019^57^	United States	Randomised Control Trial
Luft	2008^58^	United States	Randomised Control Trial
Macko	2008^59^	Italy	Pre-post trial
Macko	2005^60^	United States	Randomised Control Trial
Michalski	2023^61^	Brazil	Randomised Crossover trial
Milot	2019^36^	Canada	Randomised Control Trial
Mohd Nordin	2019^62^	Malaysia	Pre-post trial
Moore	2015^63^	United Kingdom	Randomised Control Trial
Mudge	2009^64^	New Zealand	Randomised Control Trial
Niama Natta	2021^38^	Benin, West Africa	Randomised Control Trial
Oh	2016^65^	Republic of Korea	Randomised Control Trial
Pang	2005^66^	Canada	Randomised Control Trial
Park	2016^67^	Republic of Korea	Randomised Control Trial
Pérez-De la Cruz	2020^68^	Spain	Randomised Control Trial
Quaney	2009^69^	United States	Randomised Control Trial
Raza	2021^70^	Pakistan	Non-randomised between-subjects trial
Roh	2016^71^	Republic of Korea	Randomised Control Trial
Sánchez-Sánchez	2017^37^	Spain	Randomised Control Trial
Sato	2022^72^	Japan	Randomised Control Trial
Serra	2022^73^	United States	Randomised Control Trial
Vahlberg	2017^39^	Sweden	Randomised Control Trial
Yoshioka	2022^74^	Japan	Pre-post trial

### Characteristics of sources of evidence

Table 3 presents a summary of the included reports which were conducted in various countries and predominantly randomised controlled trials. Based on information provided, despite an even split between studies of inpatients (n=16, 43%) and out-patients (n=17, 46%) sampled (studies not specified: n=4, 11%), collectively most studies were conducted in a hospital, research or rehabilitative setting (n=25, 68%), rather than in the community or home environment (n=7, 19%) or unspecified environments (n=5, 14%). Furthermore, studies used supervised exercise sessions (n=36, 97%), except one study which did not specify supervision arrangements. This suggested that participants were likely given explicit instruction within sessions and did not need to direct their own participation. Almost half of studies focused on enhancing a combination of either muscular strength, muscular endurance and/or cardiorespiratory endurance (n=18, 49%), although many focused solely on enhancing muscular strength (n=13, 35%). A small proportion of studies focused only on enhancing cardiorespiratory endurance (n=5, 14%), and one study explicitly aimed to enhance muscular endurance. Online supplemental data 4 display the main characteristics of each intervention. The duration varied between 2 weeks and 6 months with most studies incorporating multiple training sessions each week. There was a wide range of exercises administered to participants, including low-intensity exercises such as walking, cycling, Pilates and aquatic training, as well as high-intensity exercises such as circuit, strength and resistance training.

**Table 3:** Summary of the analysed studies

Descriptive		Frequency	%
Country	Korea	7	18.92
	USA	7	18.92
	Japan	3	8.11
	Brazil	2	5.41
	Canada	2	5.41
	Spain	2	5.41
	Sweden	2	5.41
	UK	2	5.41
	Australia	1	2.70
	Benin	1	2.70
	China	1	2.70
	Egypt	1	2.70
	Iran	1	2.70
	Italy	1	2.70
	Lebanon	1	2.70
	Malaysia	1	2.70
	New Zealand	1	2.70
	Pakistan	1	2.70
	Total	37	100
Study design	Randomised control trial (between-subjects)	28	75.68
	Interventions without control	5	13.51
	Randomised trial (within subjects/crossover)	2	5.41
	Non-randomised between-subjects trial	1	2.70
	Dose escalation (non-randomised trial)	1	2.70
	Total	37	100
Setting	Hospital	11	29.73
	Rehabilitation centre/clinic/facility	8	21.62
	Home/community	7	18.92
	Research centre	6	16.22
	Not specified	5	13.51
	Total	37	100
Participants	Out-patient	17	45.95
	Inpatient	16	43.24
	Not specified	4	10.81
	Total	37	100
Exercise supervision	Supervised	36	97.30
	Unsupervised	0	0
	Not specified	1	2.70
	Total	37	100
Main fitness outcome(s) (out of all studies)	Muscular strength only	13	35.14
	Muscular endurance only	1	2.70
	Cardiorespiratory endurance only	5	13.51
	Combination	18	48.65
	Total	37	100
Number of outcome measure time points post intervention	1	35	94.59
	2	2	5.41
	Total	37	100

%, percentage of reports.

### Critical appraisal within sources of evidence

Quality assessment of the reports included in this review is provided in online supplemental data 3. The methods for the Flansbjer *et al* study^33^ that were quality assessed were reported in a separate report.^34^ Most reports consistently met the criteria for appropriate randomisation and complete outcome data in randomised controlled trials and complete data, appropriate measurement and intervention administration in non-randomised studies. Areas where the reports received the lowest quality rating included a lack of comparable baseline characteristics of groups and non-blinded assessments for randomised controlled trials, as well as not accounting for confounding variables in non-randomised studies. Despite the variation in quality evaluation of reports, all reports presented clear objectives and used suitable data collection to meet their objectives.

### Results of individual sources of evidence

Information relating to findings of individual reports is shown in online supplemental data 5. The table summarises extracted information from each report and defines whether there is reported data on exercise adherence and how it is presented.

### Synthesis of results

#### Adherence definition and measurement

The characterisation and measurement of adherence differed between reports of adherence to the overall intervention and adherence to *exercise sessions* within interventions specifically (online supplemental data 4). Intervention adherence, while not always referred to as adherence, was consistently measured by comparing the numbers of participants beginning and completing interventions (ie, recording of participant numbers for preintervention and postintervention assessment), as well as descriptions of intervention drop-outs. 29 of 37 reports provided relevant adherence information, and intervention adherence ranged between 65% and 100%, with the majority of these reports (n=21) reporting at least 80% adherence. 15 reports provided reasoning for participant intervention drop-out. Reasons for intervention non-completion included individual factors (eg, insufficient time, reduced motivation, excessive fatigue from exercise and injury) and logistic factors (eg, travel arrangements and hospital discharge). Furthermore, the use of targets or cut-offs to establish adherence was infrequent. Six reports referred to the removal of participants from interventions based on what was described as ‘non-compliance’ or not attending minimum exercise sessions. ‘Non-compliance’ cut-off limits were not transparently described in reports.

Exercise session adherence was demonstrated by engagement and the level of output participants exhibited during exercise sessions. As seen in online supplemental data 4, less than half of reports (n=15) provided information describing exercise session adherence. Most of these reports (n=12) compared the number of training sessions participants attended to all organised sessions. For these reports, adherence rates varied for most participants between 83.1% and 100% (intervention groups: M=89.3%, SD=6.56). Two studies recorded hours of accumulated exercise over the course of the intervention.^35 36^ Almost half of the reports that described exercise session adherence reported the duration of exercise training completed (n=7) and almost half (n=7) reported the level of intensity during the training sessions (eg, heart rate monitoring or perceived exertion). These data were rarely reported in relation to target intensities or cut-offs to establish true adherence.

Only one report considered longer-term adherence to exercise after the intervention.^37^ While this report stated 100% (7/7 participants) intervention adherence, only the adherence rate of five of seven participants was reported (three participants attending at least 85.7% of sessions, two attended 30% of sessions, two were unreported). Additionally, only three of seven participants in the intervention group adhered to their exercise programme during the following 3-month follow-up. Furthermore, there was a second report which did not measure continued adherence to exercise training, but did assess participants’ general physical activity uptake for 3 months prior to a 4-year follow-up study.^33^ In this report, 70% of participants were physically active 3–5 days per week, and the rest were active 1–2 days per week.

#### Methods to enhance adherence

Only two studies employed methods to enhance adherence and participants’ adoption of exercise. In Niama Natta and colleagues’^38^ community-based study, the research team phoned and visited participants weekly. The authors also noted that they motivated participants to complete exercises; however, no information was provided on how they motivated participants. Vahlberg and colleagues^39^ incorporated motivational group discussions after each training session provided by a physiotherapist and assistant to help participants to fully adhere to the exercise programme. These sessions consisted of discussions relating to physical activity goals, facilitators and barriers. Their community-based study achieved full intervention adherence, as well as high attendance rates of training sessions (median=91%).

## Discussion

This systematic scoping review aimed to broadly explore adherence to cardiorespiratory and resistance training for people≥6 months after stroke. Adherence was defined and measured by two different concepts: intervention adherence (ie, retention) and exercise session adherence (compliance to exercise sessions within interventions). In general, adherence was consistently measured using intervention completion rates. By this measure, participants who were 6 months post stroke mostly adhered to interventions. However, considerably fewer studies provided information which could be used to demonstrate adherence to exercise session targets. This distinction is important because intervention adherence only describes whether participants completed the intervention or not. In contrast, exercise session adherence describes the quality of participant engagement within the programme. For example, two participants may complete an exercise intervention, but one attends half of the prescribed sessions and the other attends all sessions. It is very likely that better outcomes will be observed in the latter participant. Comparing actual exercise intensities and durations to prescribed levels also offers much more insight than intervention adherence rates. As such, a recommendation for future research is to include measures of both types of adherence (ie, intervention and exercise session adherence).

Most reports provided information regarding intervention adherence. Intervention adherence rates were found to range between 65% and 100% and most were at least 80% adherence. Using completion rate measures aligns with previous reviews in physical activity, exercise and stroke research that have called for consistent adherence measures in research^22 40^ and those that broadly define adherence as full completion of interventions.^15^ The rates imply that existing intervention designs are effective and people who experience long-term effects of stroke are largely completing exercise intervention programmes. To some degree, this is unsurprising as most studies were conducted within highly controlled environments, such as hospitals, rehabilitative centres or in research laboratories. Furthermore, even though some studies were conducted in the home or community environment, virtually all studies used supervised exercise sessions with therapists or trainers. Self-directed therapy can result in poorer adherence,^41^ and home-based training is vulnerable to adherence drop-off if it is not closely managed.^40^ Indeed, the study by Sánchez-Sánchez and colleagues^37^ was the only one to assess post intervention continued exercise engagement and found a substantial drop in exercise levels when the intervention finished. To avoid such adherence drop-off, it is recommended that future interventions cultivate robust motivation that does not require external support for exercise and include future planning for self-maintained exercise. In the current review, only two community-based studies explicitly used methods to help their participants adhere to exercise. These methods, however, seemed to be lacking in empirical support or did not specify whether they targeted the development of long-term (ie, post intervention) motivation and adherence.

Judging adherence to exercise interventions by measuring completion rates lacked the depth required to altogether understand how people engaged with exercise programmes. Unfortunately, only 15 reports (40.5%) gave detail of participants’ engagement with exercise beyond intervention adherence rates. Most of these reports recorded the number of exercise sessions participants attended, whereas a few measured the intensity or duration that exercise was sustained. These measures of adherence have been noted somewhat similarly in some previous reviews,^21 25^ though other reviews in broad physical activity and exercise contexts for stroke have found use of questionnaires, diaries and logbooks from participants to measure exercise adherence.^22 42^ It is possible that differences in exercise adherence measures identified between reviews were found because some reviews included studies involving unsupervised exercise, whereas this review coincidentally examined studies which assessed adherence by persons delivering supervised exercise interventions, even though exercise supervision was not included in the study selection criteria. Despite contextual differences between reports explored in different reviews including this one, this review has further highlighted the lack of reporting on exercise adherence and a need for standardised reporting. The relatively low number of reports documenting exercise session adherence implies that this is a widespread issue in research on poststroke exercise interventions. This conclusion would benefit from further research to confirm exercise adherence under-reporting prevalence in interventions aimed at enhancing general health-related aspects of fitness such as flexibility, balance and body composition.

Exercise session attendance rates ranged between 61% and 100%, which is comparable to other reviews in stroke rehabilitation.^18^ On the one hand, this finding implies that stroke survivors for the most part engage with exercises aimed at enhancing their physical endurance and strength. On the other hand, this variability in exercise engagement will likely influence the success of the programme. Therefore, it is important that all exercise intervention studies measure exercise adherence as a potential moderator of programme effectiveness. Other measures of exercise adherence included duration and intensity of exercise (ie, dose) that people sustained. These measures arguably could provide an even more nuanced portrait of exercise adherence, although they are more logistically demanding to measure, compared with session attendance. This likely explains why only a small number of studies included these measurements despite them providing useful information. A lack of reporting on people’ ability to meet target session durations and intensities continued to be a problem in intervention research, despite previous recommendations to do so.^21^ Moreover, it is difficult to determine how feasible higher intensity exercise programmes are for the population without such data. This issue is particularly important because there is evidence to suggest that higher intensity exercise can have greater benefit to people after a stroke.^43^ Not only would comprehensive reporting of exercise adherence ensure intervention descriptions correspond with quality assurance frameworks, such as the Consensus on Exercise Reporting Template,^43^ but would also establish the suitability of exercise programmes for people after stroke.

### Limitations

This review included reports irrespective of the judged quality as we were not looking to establish the effectiveness of interventions but to determine what was being undertaken in the field more broadly. From recommendations within the MMAT tool,^34^ it was deemed appropriate to include all reports, even though it is recognised that varying quality of reports may have impacted the overall conclusions made from the data. Additionally, data collection and analysis of reports were conducted by the main author independently, but report evaluations were checked by additional authors. It is recognised that this may have influenced the overall findings of the review. Furthermore, while this review did not exclude studies based on participants’ level of disability (but rather only the duration of time after a stroke), only including exercise programmes without physical assistance may have excluded participants with significant disability. Therefore, it is likely that any generalisation of findings from this review to people with severe disability needs to be confirmed by further research.

### Summary and conclusions

This scoping review found that there is an evident distinction between adherence to interventions and adherence to exercise session targets within interventions. It was clear that many studies did not explicitly measure adherence to exercise sessions; instead, most focused simply on intervention completion rates. Exercise session adherence information mostly concerned training session attendance rates, and employing these methods typically demonstrated high participant engagement. These rates may reflect the highly controlled and structured training environments used by nearly all studies. However, very few of these data were relative to targets or cut-off criteria. It was also apparent that there is a lack of postintervention exercise adherence measures, resulting in uncertainty as to whether people engage in exercise after the intervention. Future research should establish the quality of intervention engagement by measuring exercise session target adherence and seek to develop motivation for people after stroke to build long-term exercise routines.

## Supplementary material

10.1136/bmjsem-2024-002102 online supplemental file 1

## Data Availability

All data relevant to the study are included in the article or uploaded as supplementary information.

## References

[1] Feigin VL, Brainin M, Norrving B (2022). World Stroke Organization (WSO): Global Stroke Fact Sheet 2022. Int J Stroke.

[2] Chohan SA, Venkatesh PK, How CH (2019). Long-term complications of stroke and secondary prevention: an overview for primary care physicians. Singapore Med J.

[3] Feigin VL, Stark BA, Johnson CO (2021). Global, regional, and national burden of stroke and its risk factors, 1990–2019: a systematic analysis for the Global Burden of Disease Study 2019. Lancet Neurol.

[4] Murphy SJX, Werring DJ (2020). Stroke: causes and clinical features. Medicine (Abingdon).

[5] Lip GYH, Lane DA, Lenarczyk R (2022). Integrated care for optimizing the management of stroke and associated heart disease: a position paper of the European Society of Cardiology Council on Stroke. Eur Heart J.

[6] Veldema J, Jansen P (2021). Aquatic therapy in stroke rehabilitation: systematic review and meta-analysis. Acta Neurol Scand.

[7] Brouwer R, Wondergem R, Otten C (2021). Effect of aerobic training on vascular and metabolic risk factors for recurrent stroke: a meta-analysis. Disabil Rehabil.

[8] Veldema J, Jansen P (2020). Resistance training in stroke rehabilitation: systematic review and meta-analysis. Clin Rehabil.

[9] Saunders DH, Greig CA, Mead GE (2014). Physical Activity and Exercise After Stroke. Stroke.

[10] Kluding PM, Tseng BY, Billinger SA (2011). Exercise and executive function in individuals with chronic stroke: a pilot study. J Neurol Phys Ther.

[11] Swatridge K, Regan K, Staines WR (2017). The Acute Effects of Aerobic Exercise on Cognitive Control among People with Chronic Stroke. J Stroke Cerebrovasc Dis.

[12] Tai D, Falck RS, Davis JC (2022). Can exercise training promote better sleep and reduced fatigue in people with chronic stroke? A systematic review. J Sleep Res.

[13] Intercollegiate Stroke Working Party (2023). National clinical guideline for stroke for the UK and Ireland, London. www.strokeguideline.org.

[14] Sabaté E (2003). Adherence to long-term therapies: evidence for action.

[15] Frost R, Levati S, McClurg D (2017). What Adherence Measures Should Be Used in Trials of Home-Based Rehabilitation Interventions? A Systematic Review of the Validity, Reliability, and Acceptability of Measures. Arch Phys Med Rehabil.

[16] MacKay-Lyons M, Billinger SA, Eng JJ (2020). Aerobic Exercise Recommendations to Optimize Best Practices in Care After Stroke: AEROBICS 2019 Update. Phys Ther.

[17] Bollen JC, Dean SG, Siegert RJ (2014). A systematic review of measures of self-reported adherence to unsupervised home-based rehabilitation exercise programmes, and their psychometric properties. BMJ Open.

[18] Donoso Brown EV, Nolfi D, Wallace SE (2020). Home program practices for supporting and measuring adherence in post-stroke rehabilitation: a scoping review. Top Stroke Rehabil.

[19] Lu R, Lloyd-Randolfi D, Jones H (2021). Assessing adherence to physical activity programs post-stroke at home: A systematic review of randomized controlled trials. Top Stroke Rehabil.

[20] Mahmood A, Deshmukh A, Natarajan M (2022). Development of strategies to support home-based exercise adherence after stroke: a Delphi consensus. BMJ Open.

[21] Hawley-Hague H, Horne M, Skelton DA (2016). Review of how we should define (and measure) adherence in studies examining older adults’ participation in exercise classes. BMJ Open.

[22] Mahmood A, Nayak P, Deshmukh A (2023). Measurement, determinants, barriers, and interventions for exercise adherence: A scoping review. J Bodyw Mov Ther.

[23] Grefkes C, Fink GR (2020). Recovery from stroke: current concepts and future perspectives. *Neurol Res Pract*.

[24] Pogrebnoy D, Dennett A (2020). Exercise Programs Delivered According to Guidelines Improve Mobility in People With Stroke: A Systematic Review and Meta-analysis. Arch Phys Med Rehabil.

[25] Ammann BC, Knols RH, Baschung P (2014). Application of principles of exercise training in sub-acute and chronic stroke survivors: a systematic review. BMC Neurol.

[26] Munn Z, Peters MDJ, Stern C (2018). Systematic review or scoping review? Guidance for authors when choosing between a systematic or scoping review approach. BMC Med Res Methodol.

[27] Peters MDJ, Marnie C, Colquhoun H (2021). Scoping reviews: reinforcing and advancing the methodology and application. Syst Rev.

[28] Tricco AC, Lillie E, Zarin W (2018). PRISMA Extension for Scoping Reviews (PRISMA-ScR): Checklist and Explanation. Ann Intern Med.

[29] Caspersen CJ, Powell KE, Christenson GM (1985). Physical activity, exercise, and physical fitness: definitions and distinctions for health-related research. Public Health Rep.

[30] Veritas Health Innovation Covidence systematic review software. Melbourne, Australia. www.covidence.org.

[31] Richardson WS, Wilson MC, Nishikawa J (1995). The well-built clinical question: a key to evidence-based decisions. ACP J Club.

[32] Hong QN, Fàbregues S, Bartlett G (2018). The Mixed Methods Appraisal Tool (MMAT) version 2018 for information professionals and researchers. *EFI*.

[33] Flansbjer UB, Lexell J, Brogårdh C (2012). Long-term benefits of progressive resistance training in chronic stroke: a 4-year follow-up. J Rehabil Med.

[34] Flansbjer UB, Miller M, Downham D (2008). Progressive resistance training after stroke: Effects on muscle strength, muscle tone, gait performance and perceived participation. Acta Derm Venereol.

[35] Chun SP, Kim KY, Kang TG (2015). A Study on Core Stability Training for Postural Control Ability and Respiratory Function in Patients with Chronic Stroke. *IJBSBT*.

[36] Milot MH, Léonard G, Corriveau H (2019). Using the Borg rating of perceived exertion scale to grade the intensity of a functional training program of the affected upper limb after a stroke: a feasibility study. Clin Interv Aging.

[37] Sánchez-Sánchez ML, Ruescas-Nicolau M-A, Pérez-Miralles J-A (2017). Pilot randomized controlled trial to assess a physical therapy program on upper extremity function to counteract inactivity in chronic stroke. Top Stroke Rehabil.

[38] Niama Natta DD, Lejeune T, Detrembleur C (2021). Effectiveness of a self-rehabilitation program to improve upper-extremity function after stroke in developing countries: A randomized controlled trial. Ann Phys Rehabil Med.

[39] Vahlberg B, Lindmark B, Zetterberg L (2017). Body composition and physical function after progressive resistance and balance training among older adults after stroke: an exploratory randomized controlled trial. Disabil Rehabil.

[40] Morris JH, MacGillivray S, Mcfarlane S (2014). Interventions to Promote Long-Term Participation in Physical Activity After Stroke: A Systematic Review of the Literature. Arch Phys Med Rehabil.

[41] Yao M, Chen J, Jing J (2017). Defining the rehabilitation adherence curve and adherence phases of stroke patients: an observational study. Patient Prefer Adherence.

[42] Levy T, Laver K, Killington M (2019). A systematic review of measures of adherence to physical exercise recommendations in people with stroke. Clin Rehabil.

[43] Slade SC, Dionne CE, Underwood M (2016). Consensus on Exercise Reporting Template (CERT): Explanation and Elaboration Statement. *Br J Sports Med*.

[44] Bang DH, Son YL (2016). Effect of intensive aerobic exercise on respiratory capacity and walking ability with chronic stroke patients: a randomized controlled pilot trial. J Phys Ther Sci.

[45] Boyne P, Dunning K, Carl D (2015). Within-session responses to high-intensity interval training in chronic stroke. Med Sci Sports Exerc.

[46] Boyne P, Dunning K, Carl D (2016). High-Intensity Interval Training and Moderate-Intensity Continuous Training in Ambulatory Chronic Stroke: Feasibility Study. Phys Ther.

[47] Cramp MC, Greenwood RJ, Gill M (2006). Low intensity strength training for ambulatory stroke patients. Disabil Rehabil.

[48] Dite W, Langford ZN, Cumming TB (2015). A Phase 1 exercise dose escalation study for stroke survivors with impaired walking. Int J Stroke.

[49] El-Tamawy MS, Darwish MH, Basheer MA (2021). Effect of cycling exercise on motor excitability and gait abnormalities in stroke patients. *Egypt J Neurol Psychiatry Neurosurg*.

[50] Fathi S, Taghizadeh G, Azad A (2022). Effects of Upper Extremity Coordination Exercises Based on Fatigue Prediction on Upper Extremity Sensory-motor Functions in Chronic Stroke Survivors. *IRJ*.

[51] Fonseca GF, Midgley AW, Billinger SA (2022). Acute effects of mixed circuit training on hemodynamic and cardiac autonomic control in chronic hemiparetic stroke patients: A randomized controlled crossover trial. Front Physiol.

[52] Hashidate H, Shiomi T, Sasamoto N (2011). Effects of 6 Months Combined Functional Training on Muscle Strength, Postural Balance and Gait Performance in Community-dwelling Individuals with Chronic Stroke Hemiplegia. J Phys Ther Sci.

[53] Jin H, Jiang Y, Wei Q (2013). Effects of aerobic cycling training on cardiovascular fitness and heart rate recovery in patients with chronic stroke. NeuroRehabilitation.

[54] Kim JO, Lee J, Lee BH (2017). Effect of Scapular Stabilization Exercise during Standing on Upper Limb Function and Gait Ability of Stroke Patients. J Neurosci Rural Pract.

[55] Kim K, Lee B, Lee W (2014). Effect of Gross Motor Group Exercise on Functional Status in Chronic Stroke: A Randomized Controlled Trial. J Phys Ther Sci.

[56] Lattouf NA, Tomb R, Assi A (2021). Eccentric training effects for patients with post-stroke hemiparesis on strength and speed gait: A randomized controlled trial. NeuroRehabilitation.

[57] Linder SM, Rosenfeldt AB, Davidson S (2019). Forced, Not Voluntary, Aerobic Exercise Enhances Motor Recovery in Persons With Chronic Stroke. Neurorehabil Neural Repair.

[58] Luft AR, Macko RF, Forrester LW (2008). Treadmill exercise activates subcortical neural networks and improves walking after stroke: a randomized controlled trial. Stroke.

[59] Macko RF, Benvenuti F, Stanhope S (2008). Adaptive physical activity improves mobility function and quality of life in chronic hemiparesis. J Rehabil Res Dev.

[60] Macko RF, Ivey FM, Forrester LW (2005). Treadmill Exercise Rehabilitation Improves Ambulatory Function and Cardiovascular Fitness in Patients With Chronic Stroke. Stroke.

[61] Michalski AC, Ferreira AS, Midgley AW (2023). Mixed circuit training acutely reduces arterial stiffness in patients with chronic stroke: a crossover randomized controlled trial. Eur J Appl Physiol.

[62] Mohd Nordin NA, Yusoff NAH, Ajit Singh DK (2019). Facilitating Exercise Engagement among Community Dwelling Stroke Survivors: Is a once Per Week Group Session Sufficient?. Int J Environ Res Public Health.

[63] Moore SA, Hallsworth K, Jakovljevic DG (2015). Effects of Community Exercise Therapy on Metabolic, Brain, Physical, and Cognitive Function Following Stroke: A Randomized Controlled Pilot Trial. Neurorehabil Neural Repair.

[64] Mudge S, Barber PA, Stott NS (2009). Circuit-based rehabilitation improves gait endurance but not usual walking activity in chronic stroke: a randomized controlled trial. Arch Phys Med Rehabil.

[65] Oh DS, Park SE (2016). The effect of lumbar stabilization exercise on the pulmonary function of stroke patients. J Phys Ther Sci.

[66] Pang MYC, Eng JJ, Dawson AS (2005). A community-based fitness and mobility exercise program for older adults with chronic stroke: a randomized, controlled trial. *J Am Geriatr Soc*.

[67] Park GD, Choi JU, Kim YM (2016). The effects of multidirectional stepping training on balance, gait ability, and falls efficacy following stroke. J Phys Ther Sci.

[68] Pérez-de la Cruz S (2020). Comparison of Aquatic Therapy vs. Dry Land Therapy to Improve Mobility of Chronic Stroke Patients. IJERPH.

[69] Quaney BM, Boyd LA, McDowd JM (2009). Aerobic exercise improves cognition and motor function poststroke. Neurorehabil Neural Repair.

[70] Raza MA, Waris M, Murtaza F (2021). Effects of Treadmill Training and Stationary Cycling Training to Improve Ambulatory Function and Cardiovascular Fitness. *PJMHS*.

[71] Roh S, Gil HJ, Yoon S (2016). Effects of 8 weeks of mat-based Pilates exercise on gait in chronic stroke patients. J Phys Ther Sci.

[72] Sato C, Kamijo Y-I, Sakurai Y (2022). Three-week exercise and protein intake immediately after exercise increases the 6-min walking distance with simultaneously improved plasma volume in patients with chronic cerebrovascular disease: a preliminary prospective study. BMC Sports Sci Med Rehabil.

[73] Serra MC, Hafer-Macko CE, Robbins R (2022). Randomization to Treadmill Training Improves Physical and Metabolic Health in Association With Declines in Oxidative Stress in Stroke. Arch Phys Med Rehabil.

[74] Yoshioka K, Watanabe T, Maruyama N (2022). Two-Month Individually Supervised Exercise Therapy Improves Walking Speed, Step Length, and Temporal Gait Symmetry in Chronic Stroke Patients: A before–after Trial. Healthcare (Basel).

