# Supplementary material for: Adherence to exercise rehabilitation programmes in stroke survivors: a scoping review
Source: BMJ Open Sport Exerc Med. 2025 Sep 10;11(3):e002102. doi: 10.1136/bmjsem-2024-002102 (PMC12519362; doi:10.1136/bmjsem-2024-002102)
Supplement: online supplemental file 1 [file bmjsem-11-3-s001.pdf]

A Scoping Review of Adherence to Exercise Rehabilitation Programmes in Stroke Survivors

Supplementary File 1

Table of Contents

Supplementary Data 1: Researcher Checklist ..……………………………………………2

Supplementary Data 2: Search Strategy……………………………………………………4

Supplementary Data 3: The Mixed Methods Appraisal Tool (MMAT)………………………6

Supplementary Data 4: Descriptive Characteristics of the Included Studies………………...13

Supplementary Data 5: Participant Adherence to Studies Exercise Interventions…………...17

Supplementary Data 1

Preferred Reporting Items for Systematic reviews and Meta-Analyses extension for Scoping Reviews (PRISMA-ScR) Checklist (As per the original Manuscript)

SECTION	ITEM	PRISMA-ScR CHECKLIST ITEM	REPORTED ON PAGE #
**TITLE**			
Title	1	Identify the report as a scoping review.	1
**ABSTRACT**			
Structured summary	2	Provide a structured summary that includes (as applicable): background, objectives, eligibility criteria, sources of evidence, charting methods, results, and conclusions that relate to the review questions and objectives.	3
**INTRODUCTION**			
Rationale	3	Describe the rationale for the review in the context of what is already known. Explain why the review questions/objectives lend themselves to a scoping review approach.	4-5
Objectives	4	Provide an explicit statement of the questions and objectives being addressed with reference to their key elements (e.g., population or participants, concepts, and context) or other relevant key elements used to conceptualize the review questions and/or objectives.	5
**METHODS**			
Protocol and registration	5	Indicate whether a review protocol exists; state if and where it can be accessed (e.g., a Web address); and if available, provide registration information, including the registration number.	6
Eligibility criteria	6	Specify characteristics of the sources of evidence used as eligibility criteria (e.g., years considered, language, and publication status), and provide a rationale.	6
Information sources*	7	Describe all information sources in the search (e.g., databases with dates of coverage and contact with authors to identify additional sources), as well as the date the most recent search was executed.	6
Search	8	Present the full electronic search strategy for at least 1 database, including any limits used, such that it could be repeated.	7, Supplementary Data 2
Selection of sources of evidence†	9	State the process for selecting sources of evidence (i.e., screening and eligibility) included in the scoping review.	7
Data charting process‡	10	Describe the methods of charting data from the included sources of evidence (e.g., calibrated forms or forms that have been tested by the team before their use, and whether data charting was done independently or in duplicate) and any processes for obtaining and confirming data from investigators.	7
Data items	11	List and define all variables for which data were sought and any assumptions and simplifications made.	7
Critical appraisal of individual sources of evidence§	12	If done, provide a rationale for conducting a critical appraisal of included sources of evidence; describe the methods used and how this information was used in any data synthesis (if appropriate).	7-8

SECTION	ITEM	PRISMA-ScR CHECKLIST ITEM	REPORTED ON PAGE #
Synthesis of results	13	Describe the methods of handling and summarizing the data that were charted.	8
**RESULTS**			
Selection of sources of evidence	14	Give numbers of sources of evidence screened, assessed for eligibility, and included in the review, with reasons for exclusions at each stage, ideally using a flow diagram.	10
Characteristics of sources of evidence	15	For each source of evidence, present characteristics for which data were charted and provide the citations.	10-11, 16; Supplementary Data 4
Critical appraisal within sources of evidence	16	If done, present data on critical appraisal of included sources of evidence (see item 12).	11-12
Results of individual sources of evidence	17	For each included source of evidence, present the relevant data that were charted that relate to the review questions and objectives.	12; Supplementary Data 5
Synthesis of results	18	Summarize and/or present the charting results as they relate to the review questions and objectives.	12-14
**DISCUSSION**			
Summary of evidence	19	Summarize the main results (including an overview of concepts, themes, and types of evidence available), link to the review questions and objectives, and consider the relevance to key groups.	17-19
Limitations	20	Discuss the limitations of the scoping review process.	20
Conclusions	21	Provide a general interpretation of the results with respect to the review questions and objectives, as well as potential implications and/or next steps.	20-21
**FUNDING**			
Funding	22	Describe sources of funding for the included sources of evidence, as well as sources of funding for the scoping review. Describe the role of the funders of the scoping review.	22

JBI = Joanna Briggs Institute; PRISMA-ScR = Preferred Reporting Items for Systematic reviews and Meta-Analyses extension for Scoping Reviews.

* Where *sources of evidence* (see second footnote) are compiled from, such as bibliographic databases, social media platforms, and Web sites.

† A more inclusive/heterogeneous term used to account for the different types of evidence or data sources (e.g., quantitative and/or qualitative research, expert opinion, and policy documents) that may be eligible in a scoping review as opposed to only studies. This is not to be confused with *information sources* (see first footnote).

‡ The frameworks by Arksey and O'Malley (6) and Levac and colleagues (7) and the JBI guidance (4, 5) refer to the process of data extraction in a scoping review as data charting.

§ The process of systematically examining research evidence to assess its validity, results, and relevance before using it to inform a decision. This term is used for items 12 and 19 instead of "risk of bias" (which is more applicable to systematic reviews of interventions) to include and acknowledge the various sources of evidence that may be used in a scoping review (e.g., quantitative and/or qualitative research, expert opinion, and policy document).

From: Tricco AC, Lillie E, Zarin W, O'Brien KK, Colquhoun H, Levac D, et al. PRISMA Extension for Scoping Reviews (PRISMAScR): Checklist and Explanation. Ann Intern Med. 2018;169:467–473. doi: 10.7326/M18-0850.

Supplementary Data 2. Search Strategy

Boolean/Phrase Search Terms*	Database	Filters Applied	Search Date	Number of Records
"Stroke" OR "cerebrovascular accident" OR "CVA" OR "cerebrovascular event'" OR "cerebrovascular disorder" OR "cerebrovascular insult" OR "cerebrovascular trauma" OR "apoplexy" OR "severe stroke" OR "post-stroke" OR "stroke disabilit*" OR "intracerebral haemorrhage" OR "ICH" OR "ischemic stroke" OR "intracranial haemorrhage" OR "intracerebral hemorrhage" OR "intracranial hemorrhage" AND "rehab*" OR "recovery" OR "aftercare" OR "therap*" AND "exercise" OR "physical fitness" OR "physical conditioning" OR "physical training" OR "aerobic training" OR "cardiovascular training" OR "cardiopulmonary training" OR "cardiorespiratory training" OR "endurance training" OR "strength training" OR "balance training" OR "flexibility training"	MEDLINE (EBSCO)	'Scholarly (Peer Reviewed) Journals'<br>'Academic Journals'<br>'English'<br>'Linked full text'	22/04/23	162
			12/02/2024	231
	APA PsycINFO	'linked full-text'<br>'academic journals'<br>'empirical study'<br>'quantitative study'<br>'longitudinal study'<br>'clinical trial'<br>'follow-up study'<br>'prospective study'	22/04/23	23
			12/02/2024	25
	Web of Science	'Articles'<br>'Open access'<br>'English'<br>'early access'	22/04/23	1106
			12/02/2024 (Included date limit from previous search)	66
	Scopus	'All open access'<br>'article'<br>'journal'<br>'English'	22/04/23	1856
			12/02/2024	1994
	Cochrane trials (CENTRAL)	'English'	22/04/23	945
			12/02/2024 (Included date range between April 2023 and February 2024)	92
	CINAHL (EBSCO)	'Linked full text'<br>'Scholarly (Peer Reviewed) Journals'<br>'English'**	22/04/23	232
			**Total**	6732

* The following concepts were used in combination (“AND”):

Key concept 1: Stroke

“Stroke” OR “cerebrovascular accident” OR “CVA” OR “cerebrovascular event’” OR “cerebrovascular disorder” OR “cerebrovascular insult” OR “cerebrovascular trauma” OR “apoplexy” OR “severe stroke” OR “post-stroke” OR “stroke disabilit*” OR “intracerebral haemorrhage” OR “ICH” OR “ischemic stroke” OR “intracranial haemorrhage” OR “intracerebral hemorrhage” OR “intracranial hemorrhage”

Key concept 2: Rehabilitation

“rehab*” OR “recovery” OR “aftercare” OR “therap*”

Key concept 3: Exercise

“exercise” OR “physical fitness” OR “physical conditioning” OR “physical training” OR “aerobic training” OR “cardiovascular training” OR “cardiopulmonary training” OR “cardiorespiratory training” OR “endurance training” OR “strength training” OR “balance training” OR “flexibility training”

**Following filters were used for databases within CINAHL:

- Special limiters for eBook Collection (EBSCOhost): language – English
- ii. Special limiters for APA PsycArticles: document type – journal article; population group – human; open access, fully published.
- Special limiters for APA PsycInfo: publication type - peer-reviewed journal; document type – journal article; language – English; population group – human; open access, fully published
- Special limiters for Art & Architecture Source: publication type – journal article; document type – article;
- Special limiters for Art Index Retrospective (H.W. Wilson): Publication type – academic journal; document type – article
- Special limiters for Business Source Complete: publication type – academic journal; document type – article; language – English
- Special limiters for eBook Open Access (OA) Collection (EBSCOhost): language – English
- Special limiters for EconLit: publication type – journal article
- Special limiters for GreenFILE: publication type – academic journal; document type – article
- Special limiters for Library, Information Science & Technology Abstracts: publication type – academic journal; document type – article; language – English
- Special limiters for MEDLINE: human; language – English; English; publication type – journal article.
- Special limiters for MLA Directory of Periodicals: publication type – article; language – English
- Special limiters for MLA International Bibliography: publication type – journal article; language – English
- Special limiters for Regional Business News: document type- article
- Special limiters for SPORTDiscus with Full Text: document type – article; language – English; publication type – academic journal
- Duplicates removed

Supplementary Data 3. The Mixed Methods Appraisal Tool (MMAT)

		Screening questions (For all types)		2. Quantitative randomized controlled trials					3. Quantitative non-randomized					Reasons for "No" Response
**Lead Author**	**Year**	**S1**	**S2**	**2.1**	**2.2**	**2.3**	**2.4**	**2.5**	**3.1**	**3.2**	**3.3**	**3.4**	**3.5**	
Bang	2016	Yes	Yes	Yes	No	Yes	No	Yes						2.2 = Groups not statistically compared at baseline<br>2.4 = No report of blinded assessment
Boyne	2015	Yes	Yes	No	Yes	Yes	No	Yes						2.1 = Method of randomisation not reported<br>2.4 = No report of blinded assessment
Boyne	2016	Yes	Yes	Yes	No	Yes	Yes	Yes						2.2 = Significant differences in participant baseline characteristics
Chun	2015	Yes	Yes	No	No	Yes	No	No						2.1 = Method of randomisation not reported<br>2.2 = Groups not statistically compared at baseline<br>2.4 = No report of blinded assessment<br>2.5 = No reported adherence statistics
Cramp	2006	Yes	Yes						Yes	Yes	Yes	No	Yes	3.4 = No report of cofounders accounted for
Dite	2015	Yes	Yes						Yes	Yes	Yes	No	Yes	3.4 = No report of cofounders accounted for
El-Tamawy	2021	Yes	Yes	No	No	Yes	No	No						2.1 = Method of randomisation not reported<br>2.2 = Significant differences in participant baseline characteristics<br>2.5 = No reported adherence statistics

		Screening questions (For all types)		2. Quantitative randomized controlled trials					3. Quantitative non-randomized					Reasons for "No" Response
**Lead Author**	**Year**	**S1**	**S2**	**2.1**	**2.2**	**2.3**	**2.4**	**2.5**	**3.1**	**3.2**	**3.3**	**3.4**	**3.5**	
Fathi	2022	Yes	Yes	Yes	No	Yes	Yes	No						2.2 = Significant differences in participant baseline characteristics<br>2.5 = <80% adherence of intervention group
Flansbjer	2012 (2008)	Yes	Yes	Yes	Yes	Yes	Yes	Yes						
Fonseca	2022	Yes	Yes	Yes	Yes	Yes	No	Yes						2.4 = No report of blinded assessment
Hashidate	2011								Yes	Yes	Yes	No	Yes	3.4 = No report of cofounders accounted for
Jin	2013	Yes	Yes	No	No	Yes	No	No						2.1 = Method of randomisation not reported<br>2.2 = Groups not statistically compared at baseline<br>2.4 = No report of blinded assessment<br>2.5 = No reported adherence statistics
Kim	2017	Yes	Yes	Yes	No	Yes	Yes	No						2.2 = Significant differences in participant baseline characteristics<br>2.5 = <80% adherence of participant groups
Kim, K.	2014	Yes	Yes	Yes	No	Yes	No	No						2.2 = Groups not statistically compared at baseline<br>2.4 = No report of blinded assessment<br>2.5 = No reported adherence statistics
Lattouf	2021	Yes	Yes	No	No	Yes	No	No						2.1 = Method of randomisation not reported

		Screening questions (For all types)		2. Quantitative randomized controlled trials					3. Quantitative non-randomized					Reasons for "No" Response
**Lead Author**	**Year**	**S1**	**S2**	**2.1**	**2.2**	**2.3**	**2.4**	**2.5**	**3.1**	**3.2**	**3.3**	**3.4**	**3.5**	
														2.2 = Groups not statistically compared at baseline<br>2.4 = No report of blinded assessment<br>2.5 = No reported adherence statistics
Linder	2019	Yes	Yes	Yes	No	Yes	Yes	No						2.2 = Significant differences in participant baseline characteristics<br>2.5 = <80% adherence of control group, intervention group satisfactory
Luft	2008	Yes	Yes	Yes	No	Yes	Yes	No						2.2 = Significant differences in participant baseline characteristics<br>2.5 = <80% adherence of participant groups
Macko	2008	Yes	Yes						Yes	Yes	Yes	No	Yes	3.4 = No report of cofounders accounted for
Macko	2005	Yes	Yes	Yes	No	Yes	No	No						2.2 = Significant differences in participant baseline characteristics<br>2.4 = No blinded assessment<br>2.5 = <80% adherence of participant groups
Michalski	2023	Yes	Yes	Yes	Yes	Yes	No	Yes						2.4 = No report of blinded assessment
Milot	2019	Yes	Yes	Yes	No	Yes	No	Yes						2.2 = Significant differences in participant baseline characteristics<br>2.4 = No blinded assessment

		Screening questions (For all types)		2. Quantitative randomized controlled trials					3. Quantitative non-randomized					Reasons for "No" Response
**Lead Author**	**Year**	**S1**	**S2**	**2.1**	**2.2**	**2.3**	**2.4**	**2.5**	**3.1**	**3.2**	**3.3**	**3.4**	**3.5**	
Mohd Nordin	2019	Yes	Yes						Yes	Yes	Yes	No	Yes	3.4 = No report of cofounders accounted for
Moore	2015	Yes	Yes	Yes	No	Yes	Yes	Yes						2.2 = Significant differences in participant baseline characteristics
Mudge	2009	Yes	Yes	Yes	No	Yes	Yes	Yes						2.2 = Significant differences in participant baseline characteristics
Niama Natta	2021	Yes	Yes	Yes	No	Yes	Yes	No						2.2 = Significant differences in participant baseline characteristics<br>2.5 = <80% adherence of intervention group
Oh	2016	Yes	Yes	No	No	Yes	No	Yes						2.1 = Method of randomisation not reported<br>2.2 = Groups not statistically compared at baseline<br>2.4 = No report of blinded assessment
Pang	2005	Yes	Yes	Yes	No	Yes	No	Yes						2.2 = Groups not statistically compared at baseline<br>2.4 = No blinded assessment
Park	2016	Yes	Yes	No	No	Yes	No	No						2.1 = Method of randomisation not reported<br>2.2 = Groups not statistically compared at baseline<br>2.4 = No report of blinded assessment<br>2.5 = No reported adherence statistics

		Screening questions (For all types)		2. Quantitative randomized controlled trials					3. Quantitative non-randomized					Reasons for "No" Response
**Lead Author**	**Year**	**S1**	**S2**	**2.1**	**2.2**	**2.3**	**2.4**	**2.5**	**3.1**	**3.2**	**3.3**	**3.4**	**3.5**	
Perez-De la Cruz	2020	Yes	Yes	Yes	No	Yes	No	Yes						2.2 = Significant differences in participant baseline characteristics<br>2.4 = No report of blinded assessment
Quaney	2009	Yes	Yes	No	No	Yes	No	Yes						2.1 = Method of randomisation not reported<br>2.2 = Significant differences in participant baseline characteristics<br>2.4 = No report of blinded assessment
Raza	2021	Yes	Yes						Yes	Yes	Yes	No	No	3.4 = No report of cofounders accounted for<br>3.5 = Lack of information regarding intervention administration
Roh	2016	Yes	Yes	No	No	Yes	No	No						2.1 = Method of randomisation not reported<br>2.2 = Groups not statistically compared at baseline<br>2.4 = No report of blinded assessment<br>2.5 = No reported adherence statistics
Sanchez-Sanchez	2017	Yes	Yes	Yes	No	Yes	Yes	Yes						2.2 = Significant differences in participant baseline characteristics
Sato	2022	Yes	Yes	Yes	No	Yes	Yes	Yes						2.2 = Significant differences in participant baseline characteristics

		Screening questions (For all types)		2. Quantitative randomized controlled trials					3. Quantitative non-randomized					Reasons for "No" Response
**Lead Author**	**Year**	**S1**	**S2**	**2.1**	**2.2**	**2.3**	**2.4**	**2.5**	**3.1**	**3.2**	**3.3**	**3.4**	**3.5**	
Serra	2022	Yes	Yes	Yes	No	Yes	No	No						2.2 = Groups not statistically compared at baseline<br>2.4 = Reported unblinded assessment<br>2.5 = <80% adherence of intervention group
Vahlberg	2017	Yes	Yes	Yes	No	Yes	Yes	Yes						2.2 = Significant differences in participant baseline characteristics
Yoshioka	2022	Yes	Yes						Yes	Yes	Yes	No	Yes	3.4 = No report of cofounders accounted for

Table Code:

Screening questions (for all types)

S1. Are there clear research questions?

S2. Do the collected data allow to address the research questions?

1. Qualitative

1.1. Is the qualitative approach appropriate to answer the research question?

1.2. Are the qualitative data collection methods adequate to address the research question?

1.3. Are the findings adequately derived from the data?

1.4. Is the interpretation of results sufficiently substantiated by data?

1.5. Is there coherence between qualitative data sources, collection, analysis and interpretation?

2. Quantitative randomized controlled trials

2.1. Is randomization appropriately performed?

2.2. Are the groups comparable at baseline?

2.3. Are there complete outcome data?

2.4. Are outcome assessors blinded to the intervention provided?

2.5 Did the participants adhere to the assigned intervention?

3. Quantitative non-randomized

3.1. Are the participants representative of the target population? 3.2. Are measurements appropriate regarding both the outcome and intervention (or exposure)?

3.3. Are there complete outcome data?

3.4. Are the confounders accounted for in the design and analysis?

3.5. During the study period, is the intervention administered (or exposure occurred) as intended?

4. Quantitative descriptive

4.1. Is the sampling strategy relevant to address the research question?

4.2. Is the sample representative of the target population?

4.3. Are the measurements appropriate?

4.4. Is the risk of nonresponse bias low?

4.5. Is the statistical analysis appropriate to answer the research question?

5. Mixed methods

5.1. Is there an adequate rationale for using a mixed methods design to address the research question?

5.2. Are the different components of the study effectively integrated to answer the research question?

5.3. Are the outputs of the integration of qualitative and quantitative components adequately interpreted?

5.4. Are divergences and inconsistencies between quantitative and qualitative results adequately addressed?

5.5. Do the different components of the study adhere to the quality criteria of each tradition of the methods involve

Supplementary Data 4. Descriptive Characteristics of the Included Studies

Lead Author	Year	Country	Design	Setting	Sample size	In/out-patients	Main fitness outcome(s)	Investigational Exercise	Supervision	Programme length (duration)
Bang	2016	Republic of Korea	Randomised control trial	Rehabilitation centre	12	In-patient	Cardiorespiratory endurance	Cycle ergometry	Supervised	4 weeks (30 minutes x 5 per week)
Boyne	2015	United States	Randomised crossover trial	Hospital	19	Out-patient	Cardiorespiratory endurance	Treadmill walking	Supervised	3 weeks (30 minutes x 1 per week)
Boyne	2016	United States	Randomised control trial	Research centre	18	Out-patient	Cardiorespiratory endurance; muscular endurance	Treadmill walking	Supervised	4 weeks (25 minutes x 3 per week)
Chun	2015	Korea	Randomised control trial	Hospital	30	In-patient	Cardiorespiratory endurance; muscular strength	Core stability training	Not specified	8 weeks (30 minutes x 4 per week)
Cramp	2006	United Kingdom	Pre-post trial	Hospital	12	Out-patient	Muscular strength	Group circuit training	Supervised	6 months (16-24 sessions, maximum 2 per week)
Dite	2015	Australia	Dose escalation trial	Community	6	Out-patient	Cardiorespiratory endurance; muscular strength; muscular endurance	Multimodal exercise	Supervised	12 weeks (3 sessions per week)
El-Tamawy	2021	Egypt	Randomised control trial	Clinic	40	Out-patient	Muscular strength	Cycling	Supervised	10 weeks (60 minutes x 3 per week)
Fathi	2022	Iran	Randomised control trial	Hospital	36	In-patient	Muscular strength	Upper limb coordination exercises	Supervised	6 weeks (30 minutes x 3 per week)
Flansbjer	2012	Sweden	Randomised Control Trial 4-year follow-up	Rehabilitation centre	18 (of 24 original)	Out-patients	Muscular strength	Progressive resistance training	Supervised	10 weeks (90 minutes x 2 per week)
Fonseca	2022	Brazil	Randomised control trial	Research centre	7	In-patients	Cardiorespiratory endurance	Mixed circuit training	Supervised	2 weeks (1 per week)
Hashidate	2011	Japan	Pre-post trial	Community	22	Out-patients	Muscular strength	Combined functional training	Supervised	24 weeks (90 minutes x ½ per week)

Lead Author	Year	Country	Design	Setting	Sample size	In/out-patients	Main fitness outcome(s)	Investigational Exercise	Supervision	Programme length (duration)
Jin	2013	China	Randomised control trial	Not specified	128	In-patients	Cardiorespiratory endurance; muscular strength; muscular endurance	Cycling	Supervised	12 weeks (40 minutes x 5 per week)
Kim	2017	Republic of Korea	Randomised Control Trial	Hospital	24	In-patients	Muscular strength	Scapular stabilization exercise	Supervised	8 weeks (30 minutes x 3 per week)
Kim, K	2014	Republic of Korea	Randomised Control Trial	Hospital	28	In-patients	Muscular strength	Gross muscle group exercise	Supervised	6 weeks (50 minutes x 3 per week)
Lattouf	2021	Lebanon	Randomised Control Trial	Not specified	37	Out-patients	Muscular strength	Strength training	Supervised	4 weeks (30 minutes x 3 per week)
Linder	2019	United States	Randomised Control Trial	Not specified	48	In-patients	Muscular strength	Cycling	Supervised	8 weeks (90 minutes x 3 per week)
Luft	2008	United States	Randomised Control Trial	Rehabilitation centre	113	In-patient	Cardiorespiratory endurance; Muscular endurance	Treadmill walking	Supervised	24 weeks (40 minutes x 3 per week)
Macko	2008	Italy	Pre-post trial	Community	22	Out-patient	Muscular endurance; Muscular strength	Adaptive physical activity	Supervised & home sessions	8 weeks (60 minutes x 5 per week)
Macko	2005	United States	Randomised Control Trial	Hospital	61	In-patient	Cardiorespiratory endurance; Muscular endurance	Treadmill walking	Supervised	24 weeks (40 minutes x 3 per week)
Michalski	2023	Brazil	Randomised Crossover trial	Research centre	7	In-patient	Cardiorespiratory endurance	Mixed circuit training	Supervised	3 weeks (1 per week)
Milot	2019	Canada	Randomised Control Trial	Research centre	12	Out-patient	Muscular strength	Upper limb functional training	Supervised	4 weeks (60 minutes x 3 per week)
Mohd Nordin	2019	Malaysia	Pre-post trial	Medical centre	44	Out-patient	Muscular endurance; Muscular strength	Task-orientated circuit training	Supervised	12 weeks (90 minutes x 1 per week)
Moore	2015	United Kingdom	Randomised Control Trial	Community	40	Out-patient	Cardiorespiratory endurance; Muscular strength	Mixed training	Supervised	19 weeks (45-60 minutes x 3 per week)
Mudge	2009	New Zealand	Randomised Control Trial	Rehabilitation clinic	58	Not specified	Muscular endurance	Circuit training	Supervised	4 weeks (30 minutes x 3 per week)

Lead Author	Year	Country	Design	Setting	Sample size	In/out-patients	Main fitness outcome(s)	Investigational Exercise	Supervision	Programme length (duration)
Niama Natta	2021	Benin, West Africa	Randomised Control Trial	Community	59	Out-patient	Muscular strength	Self-rehabilitation program	Supervised	8 weeks (120 minutes x 6 per week)
Oh	2016	Republic of Korea	Randomised Control Trial	Hospital	40	In-patient	Cardiorespiratory endurance	Lumbar stabilization exercises	Supervised	8 weeks (40 minutes x 3 per week)
Pang	2005	Canada	Randomised Control Trial	Community	63	Out-patient	Cardiorespiratory endurance; Muscular endurance; Muscular strength	The FRAME programme (Circuit training)	Supervised	19 weeks (60 minutes x 3 per week)
Park	2016	Republic of Korea	Randomised Control Trial	Hospital	40	In-patient	Muscular strength; muscular endurance	Multidirectional stepping	Supervised	4 weeks (30 minutes x 5 per week)
Pérez-De la Cruz	2020	Spain	Randomised Control Trial	Not specified	40	Not specified	Muscular endurance; muscular strength	Aquatic Ai Chi	Supervised	12 weeks (45 minutes x 2 per week)
Quaney	2009	United States	Randomised Control Trial	Research centre	40	Not specified	Cardiorespiratory endurance; Muscular strength	Progressive resistive stationary bicycle training	Supervised	8 weeks (45 minutes x 3 per week)
Raza	2021	Pakistan	Non-randomised between-subjects trial	Hospital	54	In-patient	Cardiorespiratory endurance; Muscular endurance; Muscular strength	Treadmill and stationary cycling	Supervised	Not specified
Roh	2016	Republic of Korea	Randomised Control Trial	Not specified	20	Not specified	Muscular strength	Pilates	Supervised	8 weeks (60 minutes x 3 per week)
Sánchez-Sánchez	2017	Spain	Randomised Control Trial	Rehabilitation centre and home	15	Out-patient	Muscular strength	Circuit training	Supervised	12 weeks (33 sessions)
Sato	2022	Japan	Randomised Control Trial	Hospital	15	In-patient	Cardiorespiratory endurance; Muscular endurance; Muscular strength	Circuit training	Supervised	3 weeks (60 minutes x 11 per week)
Serra	2022	United States	Randomised Control Trial	Research centre	51	Out-patient	Cardiorespiratory endurance; muscular endurance	Treadmill walking	Supervised	24 weeks (15 to 50 minutes x 3 per week)
Vahlberg	2017	Sweden	Randomised Control Trial	Community	43	Out-patient	Muscular endurance; Muscular strength	Progressive resistance and balance training	Supervised	12 weeks (55 minutes x 2 per week)

Lead Author	Year	Country	Design	Setting	Sample size	In/out-patients	Main fitness outcome(s)	Investigational Exercise	Supervision	Programme length (duration)
Yoshioka	2022	Japan	Pre-post trial	Rehabilitation facility	25	In-patient	Muscular endurance; muscular strength	Individually tailored exercise (including aerobic and weight training)	Supervised	8 weeks (70 minutes x 2 per week)

Supplementary Data 5. Participant Adherence to Studies Exercise Interventions

Lead Author	Year	Intervention adherence measure?	Measure	Findings	Reasons for dropouts (If provided)	Exercise adherence measure?	Measure	Findings
Bang	2016	Yes	Participant completion rate	12/12 completed intervention (**100%**)		No		
Boyne	2015	Yes	Participant completion rate	18/19 completed intervention (**94.7%**)		Yes	Exercise tolerance (minutes at VO2 peak levels, minutes at set %HRR, treadmill speed, step count)	11/18 tolerated hardest P30 exercise protocol (**61.1%**); 15/18 tolerated mid-range exercise P60 protocol (**83.3%**). Hardest P30 protocol produced highest cardiorespiratory intensity but lower tolerance and treadmill speed than other protocols.
Boyne	2016	Yes	Participant completion rate	11/13 completed high-intensity interval training (**84.6%**); 5/5 completed moderate intensity training (**100%**)	Stopped coming (n=1), Injured at home (n=1)	Yes	Number of training sessions completed, minutes of training within sessions, training intensity	All 16 participants completed training sessions (**100%**), 13/16 completed 25 minutes training in each session (3 in HIT group could not reach session time for 1 -2 sessions). Both groups were able to increase intensity of training over weeks.
Chun	2015	No				No		
Cramp	2006	Yes	Participant completion rate	10/12 completed intervention (**83.3%**)	Unrelated illness (n=1), Did not attend minimum sessions (n=1)	Yes	Number of attended training sessions above the minimum requirements	Attended average 21/24 (SD=4) training sessions (**87.5%**) with 4 participants attending only the minimum number of sessions (16 sessions).
Dite	2015	Yes	Participant completion rate	6/6 completed (**100%**)		Yes	Hours of exercise training over 12-week period	5/6 participants achieved maximum of 85 hours exercise in 12 weeks. 1/6 participants accumulated 52.3 hours of exercise.
El-Tamawy	2021	No				No		
Fathi	2022	Yes	Participant completion rate	12/16 completed upper extremity training (**75%**); 12/14 completed control (**85.7%**)		No		
Flansbjer	2012	Yes	Participant completion rate	18/24 agreed to follow-up assessment (**75%**)		No, but measure of physical activity	General self-reported physical activity over 3 months	Around 70% of participants were physically active 3-5 days per week, otherwise 1-2 days per week.
Fonseca	2022	Yes	Participant completion rate	7/7 completed intervention (**100%**)		No		
Hashidate	2011	No				No		
Jin	2013	No				No		

Kim	2017	Yes	Participant completion rate	9/12 completed scapular stabilization exercises **(75%)**; 8/12 completed control exercise **(66.7%)**	Exercise group: Personal issue (n=3); Control group: Discharge from hospital (n=4)	No		
Kim, K	2014	No				No		
Lattouf	2021	No				No		
Linder	2019	Yes	Participant completion rate	16/17 completed forced exercise intensity **(85.7%)**; 16/20 completed voluntary exercise intensity **(80%)**; 8/11 completed educational control **(72.7%)**	FE: Unrelated injury (n=1); VE: non-compliance (n=3), recurrent stroke (n=1); ED: non-compliance (n=1), unrelated injury (n=2)	Yes	Aerobic intensities, speed of cycling	Exercise groups achieved similar intensities (60% HRR for forced exercise, 59% HRR for voluntarily paced exercise). 44% of the forced exercise and 50% of the voluntary exercise participants achieved ≥60% HRR. Cycling cadence was significantly higher for the forced intensity group (74 RPM) than the voluntary intensity group (59 RPM).
Luft	2008	Yes	Participant completion rate	37/57 completed task-repetitive treadmill exercise (**64.9%**); 34/56 stretching control **(60.7%)**	Exercise group: unrelated medical condition (n=12), recurrent stroke (n=2), noncompliance (n=6); Control: unrelated medical conditions (n=11), noncompliance (n=11)	Yes	% of attended planned training sessions	Exercise group attended **89%** of the planned sessions, control group attended **85%** of planned sessions.
Macko	2008	Yes	Participant completion rate	20/22 completed intervention **(90.9%)**	Unrelated injury (n=1), noncompliance (n=1)	Yes	% of training attendance (both group sessions and home sessions)	Participants attended **97%** of classes and there was full compliance of home exercise (**100%**).
Macko	2005	Yes	Participant completion rate	25/32 completed treadmill aerobic training **(78.1%)**; 20/29 completed low-intensity walking control **(69.0%)**	Exercise group: unrelated medical reasons (n=4), noncompliance (n=3); Control: unrelated medical reasons (n=6), noncompliance (n=3)	Yes	% training attendance, Training intensity and duration progression	Exercise group had **84%** attendance of sessions; control had **77%** attendance. Training group showed to progress exercise with averages of aerobic intensity (47% HRR to 58% HRR), training duration (12 minutes to 41 minutes) and training velocity (.48 to .75) increasing over 6-month period. Mean increase of 56%.
Michalski	2023	Yes	Participant completion rate	7/7 completed intervention **(100%)**		No		

Milot	2019	Yes	Participant completion rate	6/6 completed functional training **(100%)**; 6/6 completed strength training **(100%)**		Yes	% training attendance, training intensity measured by perceived exertion	**100%** attendance to training sessions. Both groups showed to similarly experienced training intensity suggesting similar compliance to intensity progression.
Mohd Nordin	2019	Yes	Participant completion rate	42/44 completed exercise intervention **(95.5%)**	Decided on traditional therapy (n=1), re-hospitalised (n=1)	Yes	% training attendance	Exercise adherence was **83.3%** (n=35) of attending at least 10 of 12 sessions.
Moore	2015	Yes	Participant completion rate	20/20 completed exercise intervention **(100%)**; 20/20 completed control **(100%)**		Yes	% training attendance, number of hours of exercise completed	Exercise group completed **>90%** of assessments and training sessions. Participants averaged 53 hours of exercise out of a maximum of 57 hours.
Mudge	2009	Yes	Participant completion rate	30/31 exercise intervention **(96.8%)**; 25/27 completed control **(92.6%)**	Exercise group: disinterest (n=1); control group: time (n=1), dislike of group (n=1)	Yes	Hours of exercise training	Exercise attended average of 10.8 hours and control attended 11.1 hours out of possible 12 hours of training.
Niama Natta	2021	Yes	Participant completion rate	20/28 completed exercise intervention **(71.4%)**; 29/31 completed control **(93.5%)**	Exercise group: travelled (n=6), hospitalisation (n=2); Control group: travelled (n=2)	No		
Oh	2016	Yes	Participant completion rate	37/40 completed study (unclear which experimental group had withdrawals) **(92.5%)**	Discharged from hospital (n=2), dropped out of study (n=1)	No		
Pang	2005	Yes	Participant completion rate	30/32 completed intervention and follow-up **(93.8%)**; 30/31 completed control **(96.8%)**	Exercise group: Exercise too fatiguing (n=1), unable to commit to time (n=1); control group: unable to commit to time (n=1)	Yes	% of training attendance, level of training intensity reached within sessions	Intervention group attended **81.4%** of sessions, control attended **80.4%** of sessions.<br>In the intervention group, 6 participants were unable to train above 40-50%HRR, 7 participants were able to train at 70-80%HRR at the end of the trial. Average time at target heart rate was 15 minutes.
Park	2016	No				No		
Perez-De la Cruz	2020	Yes	Participant completion rate	14/14 completed dry land therapy **(100%)**;<br>13/13 completed aquatic therapy **(100%)**;<br>13/13 completed aquatic therapy + dry land therapy **(100%)**		No		
Quaney	2009	Yes	Participant completion rate	38/40 completed study **(95%)**	Drop-out after enrolment (no reason given)	No		

Raza	2021	Yes	Participant completion rate	50/54 original participants completed interventions **(92.6%)**		No		
Roh	2016	No				No		
Sánchez-Sánchez	2017	Yes	Participant completion rate	7/7 completed functional training intervention **(100%)**; 8/8 completed control **(100%)** (prior 3-month follow-up assessment)		Yes	% training attendance, continuation of programme at home	The intervention group had 3 participants attending at least **85.7%** of sessions and 2 attended **30%.** Non-compliance was reasoned with hospital admission and family health problems.<br>The intervention group only had exercise adherence of 3 participants after the intervention before the 3-month follow-up assessment. Intensity and duration of sessions varied.
Sato	2022	Yes	Participant completion rate	7/8 original participants completed exercise intervention group **(87.5%)**; 7/7 original participants completed exercise control **(100%)**	Intervention group: lost motivation (n=1)	No		
Serra	2022	Yes	Participant completion rate	20/26 completed exercise intervention (**76.9%**); 19/25 completed stretching control (**76%**)	Time constraints (intervention n=2; control n=3), recurrent stroke (intervention n=1; control n=1), foot ulcer (intervention n=1), noncompliance (intervention n=2; control n=2)	Yes	% training attendance, time spent performing intervention, speed and incline whilst using the treadmill by the end of the intervention, heart rate goal achievement, use of rest breaks	Training attendance did not differ between groups (control vs. intervention: **82±1%** vs. **83±1%**; P=0.80; range: 75–97%), neither did total time spent performing the interventions (control vs. intervention: 2,383±36 vs. 2,416±40 minutes, P=0.63). For the intervention group, the average speed achieved on the treadmill was 2.2±0.3 mph (range: 0.8–5.0 mph) and the incline was ≤7%. Participants achieved a heart rate goal range 97.3±0.5% of the time. Two participants took rest breaks ~72% of sessions for less than five minutes long.
Vahlberg	2017	Yes	Participant completion rate	20/20 completed exercise intervention **(100%)**; 23/23 completed control **(100%)**		Yes	% training attendance rate, ability to perform exercise	Median attendance rate of intervention group was **91%.**
Yoshioka	2022	Yes	Participant completion rate	25/25 completed exercise intervention **(100%)**		No		
